# 

**Published:** 1890-12

**Authors:** 


					﻿Vol. X.
DECEMBER, 1890.
NO. 12.
THE
pOMlEDPATHlC pHY^lClAfl,
A Monthly Journal of Homoeopathic Materia Medica
and Clinical Medicine.
“ He is a freeman whom truth makes free, and all are slaves beside."
“Seek the Truth: come whence it may, cost what it will"
EDITED AND PUBLISHED BY
WALTER ML JAMES, ML D.,
AND
GEORGE H. CLARK, MI. D.
ASSISTED BY THE FOLLOWING CONTRIBUTORS:
E. T. Balch, M. D., South Bend, Wash.
Clarence Willard Butler, M. D.,-
Montclair, N. J.
Prof. Edmund Carleton, M. D., New
York.
Daniel W. Clausen, M. Dm Philada.
Stuart Close. M.D., Brooklyn.
Edward Cranch. M. D., Erie, Pa.
W. S. Gee, M. D.. Hyde Park, Ill.
Cla ken ok C. Howard, M. D.. New Y ork.
Samuel A. Kimball, M. D., Boston.
Edmund J. Lee, M. D., Former Editor,
Philada.
A. McNeil. M.D., San Francisco.
C. F. Nichols. M.D., Boston.
Mahlon PrestOn, M. D., Norristown.
George W. Sherbino, M. D., Abilene,
Texas.
C. Carleton Smith, M. D.,Philada.
Wm Stein rauf, M. D., St. Charles, Mo.
PHILADELPHIA:
No. 1125 SPRUCE STREET.
XiOaSTSOO 3W
ALFRED HEATH & CO., Sole Agents fob Great Britain,
114 Ebury Street. Eaton Square.
Subscription, $2.50, invariably in advance. Single numbers, 95 cts.
Entered at the Philadelphia Post-Office as Second-Class Mali Matter.
				

